# The Hospital Treatment of Lunacy

**Published:** 1902-11-01

**Authors:** 


### THE HOSPITAL TREATMENT OF LUNACY.

Everyone nowadays admits that the weak spot in our arrangements for the care and treatment of the insane, as regulated by existing lunacy laws, hinges upon the difficulties which surround the management of the incipient and as yet hardly certifiable cases. As things stand at present the solution of the problem, so far as the poor are concerned, seems to lie between two proposals; the one to admit mental patients to general hospitals, to be treated either along with the other patients or in special wards, and by special outpatient physicians; the other to establish special hospitals for mental diseases, to which patients so affected might be taken without certificate and without any "stigma," and from which they would afterwards be drafted to other institutions as occasion might require, or be discharged should they be restored to health.

So far as the patients are concerned—their sentiments, their dislike to go to any institution associated with madness, their willingness to go to ordinary hospitals—and also so far as concerns the prevention of any "stigma" on the relatives, there is no doubt that the first plan is ideal. But when we attempt to put such a plan into practice we are met at once with very considerable difficulties. The proposal to place incipient cases in general wards along with ordinary patients cannot, we think, be seriously considered. They would be a constant source of unrest and even danger to the other patients, while they themselves would run considerably greater risk than they would if placed where they could be more effectually guarded from the effects of sudden impulses. Yet if placed in special wards, wards provided with all the safeguards which are essential in the treatment of those who, although as yet uncertifiable, may be on the verge of an "outbreak," where is the difference between the "mad ward" of a hospital and the properly licensed asylum? Except so far as concerns the suggested advantage of being under a general rather than a mental physician, the difference is not in favour of the patient. If a man is a "raving maniac" as it is called, it perhaps matters little to him where he is, so that he is efficiently protected. But the man who "sees things," the man who doubts, the man who thinks he has committed some crime, or who is convinced that he is on the verge of making his fortune, is better off in a well-organised asylum in the open country than in a hospital ward. As for the benefit which the students would derive from the reception of early cases in hospital wards, we are inclined to think that this has by some been a little exaggerated. Doubtless such wards would be filled with very curious cases—cases full of interest to psychologists. But students must of necessity be taught by types, and in the limited space of time at their disposal they are likely to learn more from a carefully conducted series of clinical lectures at an asylum than by studying the odd and undeveloped cases in the "special ward." Such cases, indeed, are only comprehensible in light of a fuller knowledge.

Thus we are driven to the conclusion that if anything should be done in the way of the "hospital treatment" of early cases of insanity, it will best be done by the establishment of special hospitals in which not only can early cases be received without certificate, and thus without stigma, but in which curable or hopeful cases may be retained for treatment for a time even after they become fully certifiable. Such institutions would not be asylums, they would be hospitals in the truest sense of the word, yet they would be provided with all that is required to fit them for the safe treatment of acute as well as of incipient cases, and such hospitals would be useful schools. Whether the establishment of new hospitals for this purpose is necessary is quite another matter. A good deal too much has been made of the assumed superiority of the hospital physician, and, by inference, of the incapacity of the asylum superintendent, in the treatment of the bodily complications—maybe causes—of mental disease. There was possibly a time when asylum committees, in making their appointments, were apt to be more impressed by a knowledge of administrative details—boilers, washing-machines, and cost of provisions—than by scientific attainments. But there are to-day plenty of well qualified and most enthusiastic workers in the asylum service, and seeing that in any case some alteration of the law will be required, the question does seriously arise, whether some alteration of the terms of admission, some modification of the law as to certification, some wide classification by which certain asylums should become hospitals, others convalescent homes, while others again remain the inevitable asylums for the final reception of chronic incurables, would not render it unnecessary either to erect separate "hospitals for diseases of the mind" or to receive mental cases into general hospital wards.

